# Clinical study on the treatment of primary trigeminal neuralgia by robot-assisted percutaneous balloon compression

**DOI:** 10.3389/fsurg.2022.1007818

**Published:** 2022-09-12

**Authors:** Fa-yan Dong, Qi Zhan, Zheng-kai Shao, Qiang Gu, Xue-ting Gao, Bei Zhou, Lang Li, Yi-wen Ma, Xue-feng Wang, Yan-chao Liang

**Affiliations:** Department of Neurosurgery, The Fourth Affiliated Hospital of Harbin Medical University, Harbin, China

**Keywords:** trigeminal neuralgia, robot, percutaneous balloon compression, the navigation module, puncture

## Abstract

**Background:**

C-arm-guided percutaneous puncture balloon compression alone has risk factors of puncture failure, complications, and poor prognosis. Robot-assisted PBC can effectively increase the one-time puncture success rate and improve the safety of the procedure. However, evidence on the superiority of robot-assisted PBC over C-arm-guided PBC alone remains relatively limited.

**Methods:**

Retrospective analysis The clinical data of 60 patients with trigeminal neuralgia aged 60 years or older in the Department of Neurosurgery of the Fourth Hospital of Harbin Medical University from January 2021 to October 2021. There were 29 males and 31 females, and the patients’ ages ranged from 60 to 79 years, with an average of 71.63 ± 5.12 years. Two groups were divided according to the surgical method, the C-arm guidance-only group (30 cases, *n* = 30) and the robot-assisted group (30 cases, *n* = 30). The success rate of first puncture, total operation time, number of “pear-shaped” balloons, number of C-arm x-ray scans, and immediate postoperative relief rate were recorded in both groups, and follow-up was performed to evaluate the postoperative results and complications. The overall evaluation of postoperative results and complications was performed.

**Results:**

Intraoperative balloon compression was successfully completed in all 60 patients, and the first puncture success rate was higher in the robot-assisted group than in the simple C-arm group, with a significant difference between the two groups (*P *< 0.001). In terms of intraoperative balloon morphology, the number of “pear-shaped” balloons was higher in the PBC than in the C-arm-only PBC group, with a significant difference between the two groups (*P *< 0.005). The degree of immediate postoperative remission in the robotic group was 0 VAS score, which was not statistically significant in both groups (*P *> 0.05). By the final follow-up, the mean VAS score of the robot-assisted group was lower than that of the simple C-arm group, and both were statistically significant (*P *< 0.05); complications of masticatory muscle weakness or abnormal facial sensation occurred in both groups after surgery, but the number of cases in the robot-assisted group was less than that of the simple C-arm group.

**Conclusion:**

Robot-assisted PBC is better than PBC with a C-arm x-ray machine in terms of first puncture success rate, number of intraoperative balloon “pear-shaped” cases, number of C-arm x-ray scans and short-term efficacy.

## Introduction

Trigeminal neuralgia (TN) is considered to be one of the most painful diseases in human history. Current drug therapy includes antiepileptic drugs (carbamazepine, gabapentin, pregabalin, lamotrigine) and non-antiepileptic drugs (baclofen, tolcaine, pimozide, clomipramine, amitriptyline, tizanidine, proparacaine). The use of non-antiepileptic drug combinations in trigeminal neuralgia is a “first-line approach” and if non-antiepileptic drug combinations fail, antiepileptic drug combinations are sometimes considered, a “second-line approach” (1). However, as the duration of medication increases, some patients may develop oral drug tolerance or adverse drug reactions, thus making the pain not well controlled and relieved. This requires surgical treatment (2). In 1983 Mullan and Lichtor (3) described the percutaneous insertion of a Fogarty balloon catheter to compress the trigeminal ganglion and trigeminal ganglion compression became a viable treatment option. The most important advantage of percutaneous balloon compression (PBC) over other surgical treatments is that it is suitable for patients of advanced age or with underlying disease that cannot tolerate open surgery (4, 5) and although access to the foramen ovale using Hartel s sign has been widely used for decades, the complications associated with PBC can lead to serious consequences and even death. In this study, we retrospectively analyzed 60 patients with primary trigeminal neuralgia admitted to the Department of Neurosurgery of the Fourth Hospital of Harbin Medical University from January 2021 to October 2021 and underwent either conventional C-arm-guided PBC or surgical navigation and positioning planning system (referred to as robotic)-assisted PBC to compare the clinical outcomes and prognosis of the two groups in order to select The clinical outcomes and prognosis of the two groups were compared in order to select a better treatment modality.

## Materials and methods

### Clinical data

Among 60 patients, 29 were male and 31 were female, their ages ranged from 60 to 79 years, with an average of 71.63 ± 5.12 years. The randomized consent design was used to divide the patients into C-arm-guided group (30 patients, *n* = 30) and robot-assisted group (30 patients, *n* = 30), and all patients signed the informed consent form before surgery. The gender, age, disease duration, preoperative VAS score, pain area and follow-up time of patients in both groups are listed below (Table 1). Patients in both groups were diagnosed with primary trigeminal neuralgia according to clinical presentation and cranial CT or MRI.

**Table 1:** Patient’s demographic data.

Items	C-arm-guided group (*n *= 0)	Robot-assisted group (*n* = 30)	*P*
Age (years)	72.10 ± 5.33 (62–79)	71.17 ± 4.95 (62–78)	0.49
Gender			0.80
Male	15	14	
Female	15	16	
Duration of illness (months)	72.80 ± 40.65 (4–180)	80.00 ± 62.92 (4–240)	0.60
Preoperative VAS score	9.47 ± 0.82 (8–10)	9.17 ± 0.87 (7–10)	0.18
Pain area			0.60
Left	14	12	
Right	16	18	
Pain area			0.90
I	0	4	
II	18	14	
III	8	8	
IV	4	4	
Underlying diseases			0.82
Hypertension	14	15	
Cerebral infarction	7	5	
Heart disease	9	6	
Diabetes	3	1	
Other	5	3	
Surgical history			0.22
MVD	0	1	
RF	1	0	
Balloon compression	0	1	
Follow-up time (months)	5.47 ± 1.91 (2–10)	5.20 ± 2.19 (1–8)	0.62

### Inclusion criteria

1. Meet the diagnostic criteria for primary trigeminal neuralgia of the “Chinese Expert Consensus on the Treatment of Trigeminal Neuralgia”. 2. Age greater than 60 years. 3. Patients who have undergone regular treatment with drugs but have poor results or cannot tolerate the adverse effects of drugs, patients with visual analogue score (VAS) >4, patients with contraindications to MVD or refusal of craniotomy and patients with ineffectiveness or recurrence of MVD after surgery, and patients with indications for surgery and willingness to operate. 4. Normal communication, excluding patients with cognitive or mental disorders. 5. The study was approved by the Ethics Committee of the Fourth Affiliated Hospital of Harbin Medical University, and all patients had signed the informed consent form before surgery.

### Pre-operative robotic image preparation

**Figure 1:**
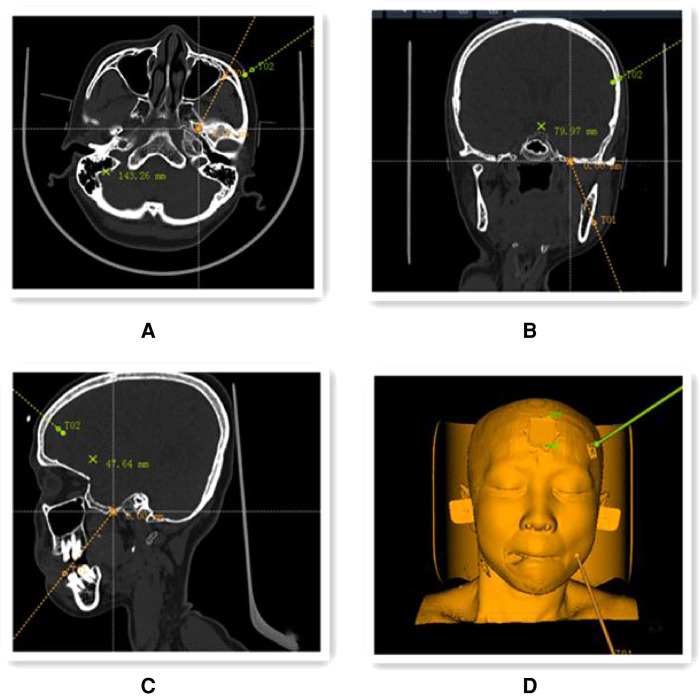
Preoperative planning using the navigation positioning planning system navigation workstation. (**A–C**) Preoperative CT and 3.0 T MRI image files were fused to select the target points of the internal foramen ovale location in coronal (**A**), axial (**B**) and sagittal (**C**) positions (yellow line in the figure shows the puncture path); (**D**) 3D reconstruction model of the head and face showing the simulated puncture path.

Robotic preoperative imaging: On the morning of the surgery, four localization marker points (Beijing Baihuiweikang Technology Co., Ltd., RM08-B type) were attached to the patient's scalp, and a cranial CT thin layer 1 mm tomography scan was performed in parallel, with the entire face within the scan area. The image files of the CT and the previous thin-layer 1 mm 3.0T MRI with medical digital imaging and communication standards were transferred to the robotic navigation workstation (Beijing Baihuiweikang Technology Co., Ltd., V4) for fusion, and the internal orifice of the foramen ovale was set as the puncture target, and the puncture path was planned on the 3D reconstruction model and CT scan (Figure 1).

### Surgical method

**Figure 2:**
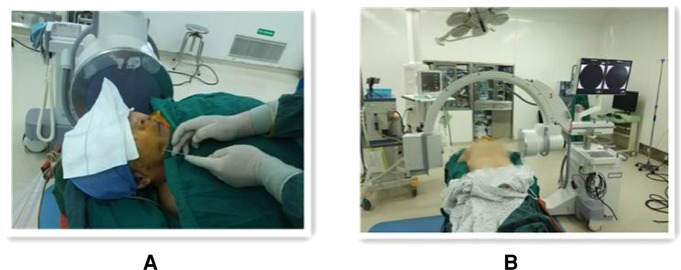
Operating procedure of percutaneous balloon compression guided by the C-arm set x-ray machine alone. (**A**) Freehand puncture with puncture needle at approximately 2.5 cm marker point next to the corner of the mouth on the painful side. (**B**) Showing the spatial layout between the patient, C-arm x-ray machine.

**Figure 3:**
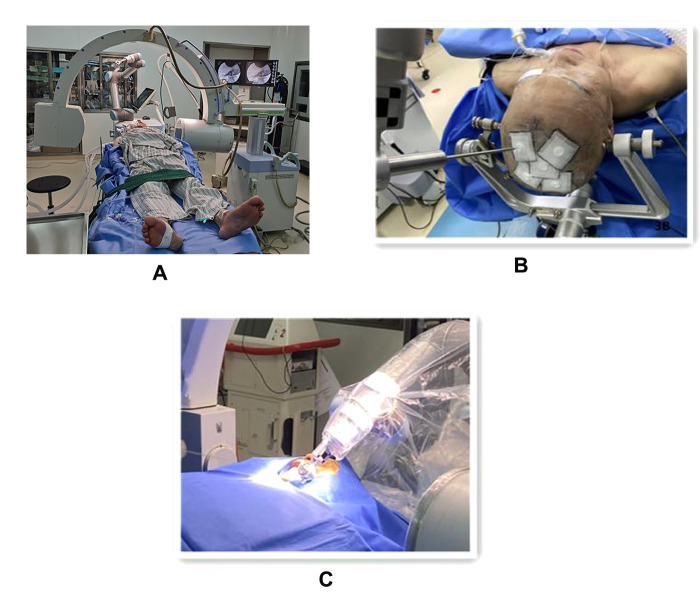
The procedure of robot-assisted percutaneous balloon compression (**A**). Showing the spatial layout between the patient, C-arm x-ray machine and robot (**B**). Showing the optical tracking locator for robotic arm and patient registration (**C**). The trocar with built-in puncture needle is punctured in the direction of the path of the instrument adapter.

**Figure 4:**
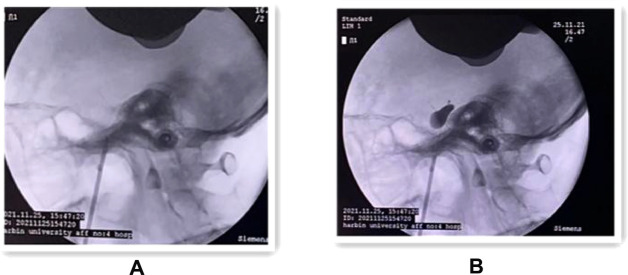
(**A**) Intraoperative lateral x-ray fluoroscopic view showing the successful puncture of the trocar into the internal orifice of the foramen ovale. (**B**) Lateral x-ray fluoroscopy showing the “pear-shaped” balloon in the filled state.

The procedure was completed under general anesthesia with the patient in the supine position, and after general anesthesia and disinfection, the Hatel anterior approach was applied, and a marker point approximately 2.5 cm next to the angle of the mouth on the painful side was selected for puncture. The C-arm alone group performed the puncture under C-arm guidance and adjusted the puncture direction and depth according to the ortho-lateral film and 3D reconstruction results until the tip of the puncture needle entered the foramen ovale (Figure 2). Patients in the robot-assisted group underwent preoperative cranial CT and 3.0T MRI imaging, followed by DICOM image reading, image fusion and segmentation, post-processing of 3D reconstruction, and loading of preoperative plans using the navigation surgical system equipped with the surgical robot “Remebot” (Beijing Baihuiweikang Technology Co., Ltd., RM100), and fixed head position in the Mayfield head frame during surgery. The bed height and the position of the special instrument trolley were adjusted, and the special instrument trolley and the Mayfield head frame were fixed with the head frame connection system. The optical tracking locator performs robotic arm and patient registration, and the robotic arm is automatically positioned for navigation based on the planned target and path and connected to the instrument adapter. Repeated reviews are performed to ensure that the adapter does not touch the buccal surface. A disposable balloon catheter kit for neurosurgery is prepared, and the cannula with the built-in puncture needle is inserted in the direction of the path of the instrument adapter until the caudal end of the cannula is attached to the adapter, at which point the head end of the cannula is located at the planned depth, at the target point of the foramen ovale (Figure 3). The head end of the balloon was located at the trigeminal foramen and the balloon was injected with 0.5–0.8 ml of contrast agent (iodophoresis), and the lateral x-ray showed a “pear-shaped” or “pear-like” balloon in the filled state (Figure 4). The total duration of balloon compression was 2.5–3.0 min before withdrawing the contrast agent, withdrawing the trocar and balloon, and applying a sterile patch to the puncture site.

### Efficacy evaluation and follow-up

The success rate of the first puncture, the number of C-arm x-ray scans, the total operative time (the time between the successful adjustment of the device after general anesthesia and the end of the operation when the puncture needle was removed), and the number of cases of intraoperative balloon “pear-shaped” were recorded, and the preoperative and postoperative conditions were assessed immediately. The preoperative and postoperative facial pain was evaluated using the visual analogue score (VAS) pain score. 1–3 VAS scores were considered excellent, 4–6 good, and 7–10 poor (6); facial numbness was evaluated using the barrow neurological institute (BNI) numbness score (7), in which grade I was no numbness and was scored as 1. Grade II is mild numbness with 2 points; Grade III is moderate numbness with some impact on life with 3 points; Grade IV is severe numbness, which is intolerable and seriously affects life and work with 4 points.

### Statistical analysis

The variables were expressed using absolute values and frequencies (for categorical variables). IBM SPSSV26.0 software was used for processing, and the measurement data were expressed as mean ± standard deviation (x ± s) along with minimum and maximum using *t*-test, and *P *< 0.05 was considered statistically significant difference.

For sample size,there are formal calculations could be performed beforehand (8). Based on the pre-experiment, the first puncture success rates were assumed to be 31% and 69% for the C-arm-only group and the robot-assisted group, respectively. The handle 1 − *β* = 0.80 and the test level *α* = 0.05 were used for sample size estimation using PASS 2021 software according to the following equation, where *p*_1_ and *p*_2_ are the estimated first puncture success rates for the C-arm-only group and the robot-assisted group, respectively, and *p* is the combined rate of the two groups.


$$n_{1}=n_{2}=\frac{{\left[u_{\alpha }\sqrt{2p(1-p)}+u_{\beta }\sqrt{\;p_{1}(1-\;p_{1})+\;p_{2}(1-\;p_{2})}\right]}^{2}}{{\left(\;p_{1}-\;p_{2}\right)}^{2}}$$

Based on the pre-defined parameters, the first puncture success rate was calculated using the “Tests for Two Proportions” menu of the PASS 2021 software for both groups, and a sample of 24 cases was required for each group. Considering a 20% lost-to-review rate, a total of 60 cases were expected to be included in this study. Thirty cases were included as the C-arm-only group and 30 subjects were included as the robot-assisted group to ensure the accuracy and scientific validity of the study results.

## Result

### Comparison of intraoperative conditions between the two groups of patients

All 60 patients successfully completed the PBC surgery, only 8 (26.7%) of the 30 patients in the simple C-arm group had a successful first puncture, and the remaining 22 (73.3%) required repeated adjustment of the needle tip position to enter the foramen ovale; in the robot-assisted group of 30 patients, 26 (86.7%) had a successful first puncture with robot assistance, and only 4 (13.3%) needed to adjust the robotic arm position again, and there was a significant difference between the two groups (*P *< 0.001). In terms of the number of C-arm machine x-ray scans, 33.03 ± 4.87 in the C-arm-only group and 19.00 ± 3.75 in the robot-assisted group, with a significant difference between the two groups (*P *< 0.001). In terms of intraoperative balloon morphology, only 14 cases (46.7%) in the C-arm-only group had ideal “pear-shaped” balloons, while 23 cases (76.7%) in the robotic-assisted group had “pear-shaped” balloons, with a significant difference between the two groups (*P *< 0.005). In terms of total operative time, the mean time in the robot-assisted group was 44.53 ± 11.43 min, which was significantly higher than that in the C-arm-only group of 19.13 ± 4.55 min (*P* < 0.001) (Table 2).

**Table 2:** Comparison of intraoperative conditions of two groups of patients.

Items	C-arm-guided group (*n* = 30)	Robot-assisted group (*n* = 30)	*P*
Number of successful first punctures			
Yes	8 (26.7%)	26 (86.7%)	<0.001
No	22 (73.3%)	4 (13.3%)	<0.001
Number of C-arm machine x-ray scans	33.03 ± 4.87 (22–39)	19.00 ± 3.75 (12–26)	0.60
Number of intraoperative balloon “pear-shaped” cases	** **	** **	0.02
Pear-shape	14 (46.7%)	23 (76.7%)	** **
No pear-shape	16 (53.3%)	7 (23.3%)	** **
Total duration of surgery (min)	19.13 ± 4.55 (12–27)	44.53 ± 11.43 (30–80)	<0.001

### Comparison of postoperative results between the two groups

The follow-up time for the balloon compression-only group ranged from 2 to 10 months, with a mean of 5.5 months; the follow-up time for the robot-assisted group ranged from 1 to 8 months, with a mean of 5.2 months, and the *P* value for the two groups was 0.62 (*P* > 0.05, not statistically significant). In the balloon compression alone group, 27 patients (90%) had immediate postoperative relief and VAS pain score decreased to 0.73 ± 2.24, and all patients had postoperative numbness on the ipsilateral side of the operation; in the robotic-assisted group, 29 patients (96.7%) had immediate postoperative relief and VAS pain score decreased to 0.03 ± 0.18. No postoperative vascular injury, diplopia, ulcerative keratitis, or infection complications occurred in either group, but patients in both groups developed ipsilateral facial numbness after surgery. By the final follow-up, the VAS evaluation of patients in the balloon compression-only group was excellent in 23 cases (76.7%), good in 6 cases (20%), and poor in 1 case (3.3%), with a VAS score of 2.20 ± 1.21 and no pain recurrence, and BNI numbness grading, grade IV in 4 cases (13.3%), grade III in 8 cases (26.7%), grade II in 18 cases (60%), with 10 cases (33.3%) masticatory muscle weakness, 6 cases (20%) had ipsilateral sensory abnormalities; 30 cases (100%) of patients in the robot-assisted group had excellent VAS evaluation, with a VAS score of 1.27 ± 0.52, and BNI numbness grading, 4 cases (13.3%) were grade IV, 6 cases (20%) were grade III, 18 cases (60%) were grade II, 2 cases (6.7%) were grade I, and there were 4 cases (13.3%) had masticatory muscle weakness and 2 cases (6.7%) had ipsilateral sensory abnormalities (Table 3).

**Table 3:** Comparison of postoperative effects of two groups of patients.

Items	C-arm-guided group (*n* = 30)	Robot-assisted group (*n* = 30)	*P*
Number of immediate postoperative remissions			0.30
Yes	27 (90%)	29 (96.7%)	
No	3 (10%)	1 (2.3%)	
Immediate postoperative VAS	0.73 ± 2.24 (0–8)	0.03 ± 0.18 (0–1)	0.10
VAS evaluation at the last follow-up			0.02
Excellent	23 (76.7%)	30 (100%)	
Good	6 (20%)	0 (0%)	
Poor	1 (3.3%)	0 (0%)	
VAS at the last follow-up	2.20 ± 1.21 (1–5)	1.27 ± 0.52 (1–3)	<0.001
**BNI** at the last follow-up			0.52
I	0 (0%)	2 (6.7%)	
II	18 (60%)	18 (60%)	
III	8 (26.7%)	6 (20%)	
IV	4 (13.3%)	4 (13.3%)	
Number of complications at the last follow-up			0.03
Number of complications at the last follow-up	10 (33.3%)	4 (13.3%)	
Sensory abnormalities	6 (20%)	2 (6.7%)	
None	14 (46.7%)	24 (80%)	

## Discussion

TN is one of the common neurological disorders whose pathogenesis is not yet clear, and although there are more treatment methods, Wang Lockliang et al. (9) concluded that a radical cure still cannot be achieved. The classical drugs for the treatment of TN are represented by sodium channel blockers such as carbamazepine or oxcarbazepine, which are suitable for mild and initial cases, and for patients who fail numerous drug treatments and are unwilling to undergo craniotomy or do not have the conditions for craniotomy, percutaneous interventions such as PBC, percutaneous radiofrequency ablation or PBC, percutaneous radiofrequency ablation, or other chemical disruptions are required to relieve pain. In particular, PBC is effective in the treatment of primary trigeminal neuralgia, and this method has the advantages of high efficiency, low trauma, and low risk. Wang Ding et al. (4) showed that percutaneous puncture balloon compression could be the preferred surgical treatment for elderly patients with primary trigeminal neuralgia over 70 years of age with average underlying conditions.

However, during PBC surgery, many serious complications such as carotid cavernous sinus fistula, dural arteriovenous fistula, subarachnoid hemorrhage, intracranial hematoma, blindness from optic nerve injury, hearing loss, up to 5% infection rate and other diseases and even death can occur due to ectopic or too deep placementof the trocar or puncture needle (10–20). In addition, it has been reported that the anatomical position, shape, and pressure of the balloon are key factors in determining the success of the procedure or the risk of complications (21), and Lai, H. L. et al. reported that the main causes of poor intraoperative balloon shaping are deviation of the puncture angle, protrusion of the needle too deeply into the anterior pontine pool, intraoperative balloon catheter drift, failure of the balloon catheter to enter the Meckel’s cavity accurately; Meckel ‘s cavity structural variants, such as bone defects and intracavitary bone spines, deeper trigeminal nerve indentation, local adhesions; rupture of the balloon too close to the puncture trocar needle and excessive pressure with restricted balloon expansion,however, 46.1% were due to deviations in puncture angle (22). However, simple lateral fluoroscopy does not adequately show the morphology of the foramen ovale, and some reports have shown that this leads to an increased rate of puncture failure in 4% of patients (23).

However, most surgeons rely only on manual or clinical experience to perform puncture and simple lateral x-ray fluoroscopy to determine the position of the balloon, which makes it difficult for even experienced surgeons to accurately grasp the angle of the puncture needle and the depth of balloon placement. For these reasons, new imaging techniques and surgical equipment are constantly being used in PBC surgery to minimize the failure rate. The use of biplane fluoroscopic imaging equipment can greatly simplify the procedure and allow for unrestricted identification of the foramen ovale during surgery. With the development of medical imaging technology, many medical institutions use Dyna-CT to guide the PBC procedure to be performed (24–26). The success rate of puncture can be greatly improved by preoperative reconstruction of the foramen ovale using 3DSlicer software, simulated puncture, measurement of relevant parameters, and intraoperative CT reconstruction (26). However, in practice, it has been found that despite the use of Dyna-CT guidance and Hartel positioning, the insertion point and direction of the needle need to be manually controlled, and puncture difficulties still exist, requiring repeated scans and punctures during the procedure (27). In addition the application of 3D CT in PBC surgical planning, guidance, and evaluation increases the radiation dose and the time of exposure under radiation relative to x-ray fluoroscopy, which may adversely affect the surgeon and the patient (28).

Currently, neurosurgical navigation techniques are reported to be the optimal solution for achieving precise puncture of the foramen ovale, which can assist in determining anatomical location and guiding surgical operations intraoperatively (29). Jordi Pérez-Bovet et al. (30) summarized all of the MEDLINE database of Pubmed involving percutaneous puncture (PR) guided by frameless neuronavigation for trigeminal neuralgia references, reported remission rates of 83%–100% after frameless neuronavigation-guided PBC, but there were significant differences in recurrence rates at follow-up, with 9.8%–64% over 1.4–5 years of follow-up. Postoperatively, 1.5%–19% had hyperalgesia and 6.2%–10.8% had masticatory muscle weakness, and only one other complication was reported with postoperative loss of corneal sensation. In a paper comparing navigation-guided vs. non-navigation PBC, Chen Shuping and colleagues (31) reported a puncture success rate of 98.15% in the navigation group and 82.24% in the non-navigation PR control group, with complications in both groups. In our study, complications occurred in 53.3% of the C-arm-only group and 20% of the robot-assisted group by the last follow-up, which was significantly less in the robot group than in the C-arm-only group, probably related to poor balloon formation due to deviations in puncture angle, as balloon shape is one of the key factors affecting complications, but due to the small number of cases and short follow-up time, it may not necessarily represent the final result (16). However, the surgery guided by purely optical and electromagnetism systems still relies on the operator's unaided operation and lacks a fixed frame constraint, whereas the robot assisted PBC surgery applied in this study has a robotic arm and adapter that can constrain the puncture needle, which can guarantee the accuracy of the needle entry angle and puncture position and ensure one-time puncture of the foramen ovale, which can avoid repeated punctures in human operation, It can avoid repeated punctures, too deep punctures and damage to intracranial structures by the needle tip. It also reduces the radiation dose and exposure time of the operator and the patient to radiation. Zheng Maohua et al. (32) also reported that for surgical beginners the use of stereotactic and neuronavigation for oval foramen puncture significantly reduced the incidence of postoperative complications in patients with trigeminal neuralgia. In addition, this technique requires a steep learning curve, and the operator's level of surgical performance can plateau after 40 procedures using the Hartel puncture method. However, robotic-assisted PBC is a safer and simpler surgical method, and its precise puncture point, puncture angle, and placement depth make it less demanding on the operator's hand, making it easier for junior surgeons and primary care providers to perform the procedure. The past decade has seen remarkable progress in the acceptance and promotion of robotics (33, 34). For example, several small case series have investigated the efficacy and safety of the Synaptive Modus V endoscopic system in spinal and cranial surgery with encouraging results (35). The 2021 study by Mallereau et al. provides the largest sample size of robotic surgery cases to date (*n* = 526). Their conclusions showed that robotic biopsies effectively increased diagnostic yields and reduced morbidity and mortality compared to traditional methods (36). That study provides evidence to confirm that robotic surgery lives up to its promises of increased safety, accuracy, and reliability.

In this study, 8 cases in the C-arm-only group were successfully punctured once, and repeated adjustments were required in most cases, 26 cases in the robot-assisted group were successfully punctured once, and the remaining 4 cases were successfully punctured after adjusting the robotic arm once, which is low compared with the 100% success rate reported by Ma Jun et al. (29) Under the assumption that the planned path is accurate, deformation of the soft tissue during needle puncture can lead to drifting of the puncture target or bending of the puncture needle due to force, both of which are the main causes of puncture accuracy (37). Although the robot in this study had a fixed robotic arm, it still could not completely overcome the interaction force that would occur between the puncture needle and the soft tissue, which may be the main reason why the other two cases in this study were not successfully punctured at once, and could be successfully punctured after readjusting the angle of the robotic arm once again, but the procedure took longer because of the need to remove and reinstall the adapter. In terms of balloon shape, the robot-assisted group had significantly more “pear-shaped” cases than the C-arm-only group, which may be related to the fact that the fixation of the robotic arm overcame the deviation of the puncture angle as the main influencing factor, but the robot could not overcome other factors that caused poor formation. Due to the reduction in the number of punctures, the number of x-ray scans in the robotic group was significantly less than that in the C-arm-only x-ray group, which also reduced the level of radiation to patients and operators. All patients in the robot-assisted group were in remission after surgery, and only one case in the simple C-arm group was not in remission; the difference between the two groups was not significant and not statistically significant (*P *> 0.05), but the mean VAS score in the robot-assisted group at the last follow-up was lower than that in the simple C-arm group, and both were statistically significant (*P *< 0.05). None of the patients in both groups had pain recurrence, which may be related to the relatively short follow-up period. Long-term follow-up data are required to reflect the actual recurrence rate, and complications and long-term prognosis also need to be observed in further follow-up. In addition, the total time of robot-assisted PBC surgery was significantly longer than that of conventional PBC, mainly because of the cumbersome preparation steps and the occasional intraoperative failure to repeat the installation of the adapter due to a single puncture would also prolong the surgery time and thus the anesthesia time of the patient. On the other hand, the optical registry cannot be fixed, and if the optical registry is touched before registration is completed, resulting in displacement, it can also increase the failure rate of the procedure.

In China, PBC was first developed by Ma Yi et al. for the treatment of PBC, but this technique has not been fully implemented in China until recent years, mainly because of the difficulty of puncturing the foramen ovale and how to adjust the ideal balloon shape. Compared with traditional PBC, robotic-assisted PBC is better than simple C-arm x-ray PBC in terms of first puncture success rate, number of intraoperative balloon “pear-shaped” cases, number of C-arm x-ray scans, and short-term efficacy, which can help improve the safety of surgery and reduce the radiation level of patients and operators. However, it has the disadvantages of long operation time and the inability to fix the optical registry, which need to be improved. However, due to the small number of cases, the follow-up time is short. Further conclusions regarding the long-term prognosis and complications need to be drawn by increasing the sample size and further follow-up observations.

## Contributions to the field statement

Trigeminal neuralgia is considered to be one of the most painful diseases in human history. For many patients who have failed drug therapy and are unwilling to undergo craniotomy or do not have the conditions for craniotomy, percutaneous interventional treatment is required, among which PBC is effective in treating primary trigeminal neuralgia. However, because the simple C-arm guided PBC procedure only relies on hand feeling or clinical experience to perform the puncture, complications associated with PBC can occur and lead to serious consequences, even death. With the development of artificial intelligence, the gradual integration of computer technology into medical devices, computer and robot-assisted surgery is a hot topic of research in the world of medicine. In this study, we compared robot-assisted trigeminal nerve balloon compression with traditional surgery and demonstrated that robot-assisted PBC is superior to PBC with C-arm x-ray machine alone in terms of first puncture success rate, number of intraoperative balloon “pear-shaped” cases, number of C-arm x-ray scans, and short-term efficacy, as well as reducing complications of surgery. It also reduces the radiation dose and exposure time of the operator and the patient, which helps to improve the safety of the operation and has a more important clinical application.

## Data Availability

The original contributions presented in the study are included in the article/Supplementary Material, further inquiries can be directed to the corresponding author/s.
